# A specific tripartite tricarboxylate transporter is involved in alginate utilization by *Vibrio* sp. C42

**DOI:** 10.1128/aem.00368-26

**Published:** 2026-06-16

**Authors:** Xiao-Meng Sun, Xiao-Han Wang, Zhao Xue, Peng Wang, Long-Sheng Zhao, Jing-Ping Wang, Ping-Yi Li, Fang Zhao, Shou-Jin Fan, Yu-Zhong Zhang, Shu-Yan Wang, Yu-Qiang Zhang, Fei Xu

**Affiliations:** 1 Marine Biotechnology Center, State Key Laboratory of Microbial Technology, Shandong University 520252, Qingdao, China; 2 Ministry of Education Key Laboratory of Evolution and Marine Biodiversity, Frontiers Science Center for Deep Ocean Multispheres and Earth System & College of Marine Life Sciences, Ocean University of China 12591, Qingdao, China; 3 Life Science College, Shandong Normal University 47856, Jinan, China; Georgia Institute of Technology, Atlanta, Georgia, USA

**Keywords:** alginate, alginate lyase, alginate utilization pathway, tripartite tricarboxylate transporter (TTT), *Vibrio*

## Abstract

Alginate is an important component of the cell wall of brown algae and is mainly utilized by bacteria in the oceans. Although many marine *Vibrio* strains are known alginate utilizers, their mechanisms for alginate utilization have not been fully understood. Here, we investigated the alginate utilization pathway of the marine bacterium *Vibrio* sp. C42, an alginate lyase-excreting strain collected from the surface of *Sargassum*. Based on genomic, transcriptomic, and proteomic analyses, an alginate utilization locus (AUL) responsible for alginate utilization was identified in *Vibrio* sp. C42. In the alginate utilization pathway, eight extracellular alginate lyases break down alginate into oligosaccharides. These oligosaccharides are then internalized through an outer membrane porin and two inner membrane transporters: sodium-solute symporter (SSS) and tripartite tricarboxylate transporter (TTT). Biochemical and structural analyses demonstrated that the TTT is novel and specific to alginate oligosaccharides. Moreover, bioinformatic analysis suggests that TTT is widely involved in alginate utilization of marine bacteria. These findings reveal a new TTT-involved alginate utilization pathway in marine *Vibrio* and other bacteria, shedding light on alginate metabolism in the sea.

**IMPORTANCE:**

Due to the huge quantity of brown algae in global oceans, alginate is an important organic carbon source in marine ecosystems. Alginate is utilized mainly by bacteria, becoming an important component of marine carbon cycling. Marine *Vibrio* strains play an important role in marine alginate utilization. Here, we reveal a novel TTT (tripartite tricarboxylate transporter) involved in the alginate utilization pathway in marine *Vibrio* sp. C42. Moreover, the TTT was identified as a novel protein specific to the transport of alginate oligosaccharides. Bioinformatic analysis further indicated its universality in the AULs of marine *Vibrio* and other bacteria, suggesting that TTT is widely involved in alginate utilization by marine bacteria. Therefore, this TTT-dependent alginate utilization pathway may have significance in marine alginate metabolism and cycling.

## INTRODUCTION

Alginate is an acidic linear polysaccharide present in great abundance in the cell wall of various brown algae, accounting for 30–60% of their dry weights (1). Alginate is composed of β-D-mannuronate (M) and its C5 epimer, α-L-guluronate (G), which are linked by 1,4-O-glycoside bonds and arranged in block structures, including homopolymeric G blocks, M blocks, and heteropolymeric MG (GM) blocks (2). Due to the huge quantity of brown algae in global oceans, alginate is an important organic carbon source in marine ecosystems utilized mainly by bacteria, becoming an important component of marine carbon cycling.

Currently, two types of alginate utilization pathways in marine bacteria have been reported. One type is adopted by only *Sphingomonas* of Proteobacteria (3). In the *Sphingomonas* strains*,* alginate-binding proteins on the cell surface concentrate alginate around a mouth-like pit that incorporates alginate polymer into the periplasm. In the periplasm, substrate-binding proteins bind alginate and pass it to an ATP-binding cassette (ABC) transporter localized in the cytoplasmic membrane (4). Once transferred into the cytoplasm, alginate is degraded by several intracellular alginate lyases to unsaturated monosaccharides. The other type is commonly used by *Flavobacteriaceae* and *Pseudoalteromonas* (5, 6). In these bacteria, alginate lyase genes and other genes involved in alginate utilization, such as transporter genes and regulator genes, are clustered in an alginate utilization locus (AUL), a specific type of polysaccharide utilization locus (PUL) dedicated to alginate degradation (7). In this pathway, extracellular alginate lyases decompose alginate to oligomers, which are then transported to the periplasm via outer membrane proteins such as SusC/SusD. Oligomers are further degraded into lower degree oligomers by alginate lyases in the periplasm (8). The resultant oligomers enter the cytoplasm through inner membrane alginate oligosaccharide transporters such as MFS system and are degraded into unsaturated monosaccharides by intracellular alginate lyases (8). In at least two pathways, intracellular unsaturated monomers are converted to 4-deoxy-L-*erythro*-5-hexoseulose uronic acid (DEH) by pectin degradation protein (KdgF) (9). DEH is reduced to 2-keto-3-deoxy-D-gluconate (KDG) by DEH reductases (DehR) (5). KDG is further phosphorylated by 2-dehydro-3-deoxy-gluconokinase (KdgK) to 2-keto-3-deoxy-6-phosphogluconate (KDPG), which is converted to pyruvate and glyceraldehyde-3-phosphate (G3P) via 2-dehydro-3-deoxy-phosphogluconate aldolase (Eda) (8). Pyruvate and G3P are catabolized in the glycolysis pathway.

*Vibrio* species are heterotrophic bacteria ubiquitous in a wide variety of marine habitats, such as seawater (10), marine animals (10), and algal samples (11). *Vibrio* bacteria are important players in marine alginate utilization. Many *Vibrio* strains that can degrade and utilize alginate have been isolated, and a few studies have investigated the alginate-utilizing mechanism of *Vibrio*. In some *Vibrio* strains, alginate utilization-associated genes are scattered throughout the genome (12). In other *Vibrio* strains, alginate utilization-associated genes are clustered in one or more AULs (7). Based on bioinformatic analysis of 25 alginate-degrading *Vibrio* strains, 21 have AULs, and four have scattered alginate degradation-associated genes, suggesting that both systems are adopted by *Vibrio* strains and that the AUL system may be more common (7). Based on gene annotation, the *Vibrio* AULs contain genes encoding multiple alginate lyases, genes encoding enzymes involved in unsaturated monosaccharide utilization (KdgF, DehR, KdgK, and Eda), and genes encoding proteins involved in transportation and regulation (7). Current research on AULs in *Vibrio* primarily focuses on how alginate lyases degrade alginate (7, 12–15). However, to date, the functions of other genes involved in alginate utilization in *Vibrio* AULs have rarely been studied. The mechanisms of transportation and regulation of alginate utilization by *Vibrio* are elusive. Thus, the alginate-utilizing mechanism of *Vibrio* needs further elucidation.

The tripartite tricarboxylate transporter (TTT) system, initially discovered in *Salmonella thyphimurium* (16), is typically composed of three components: TctA, a membrane protein with 12 transmembrane domains containing highly conserved motifs (17); TctB, a membrane protein with four transmembrane domains exhibiting lower conservation than TctA (17); and TctC, a periplasmic-binding receptor protein that typically forms dimers (18). For a TTT system, TctC binds substrates via its Venus flytrap motif, which are then transported into the cell by TctA and TctB (18). It has been reported that the TTT family transporters are capable of transporting fluorocitrate, citrate, isocitrate, cis-aconitate (16), and, more recently, D-glucarate (19). However, to date, no TTT family transporter has been reported to be involved in alginate utilization.

Here, we report a novel TTT in the AUL of *Vibrio* sp. C42, which is specific to alginate oligosaccharides and involved in alginate utilization. *Vibrio* sp. C42 is an alginate lyase-excreting strain isolated from the surface of a *Sargassum* sample collected from Shandong Province, China (20). Through genomic, transcriptomic, and proteomic analyses, we identified a complete AUL in *Vibrio* sp. C42. Biochemical analysis revealed its mechanism for alginate degradation and utilization by a variety of extracellular and intracellular enzymes. Genetic analysis suggested that extracellular degradation products were transported into the cell via a porin in the outer membrane and sodium-solute symporter (SSS) and TTT in the inner membrane. The TTT was first found in an AUL and was identified to be specific to alginate oligosaccharides by structural and biochemical analyses. Bioinformatic analysis further revealed the widespread nature of TTT within the AULs of marine bacteria.

## RESULTS AND DISCUSSION

### Analysis of the AUL and scattered alginate lyase genes in *Vibrio* sp. C42

**Fig 1:**
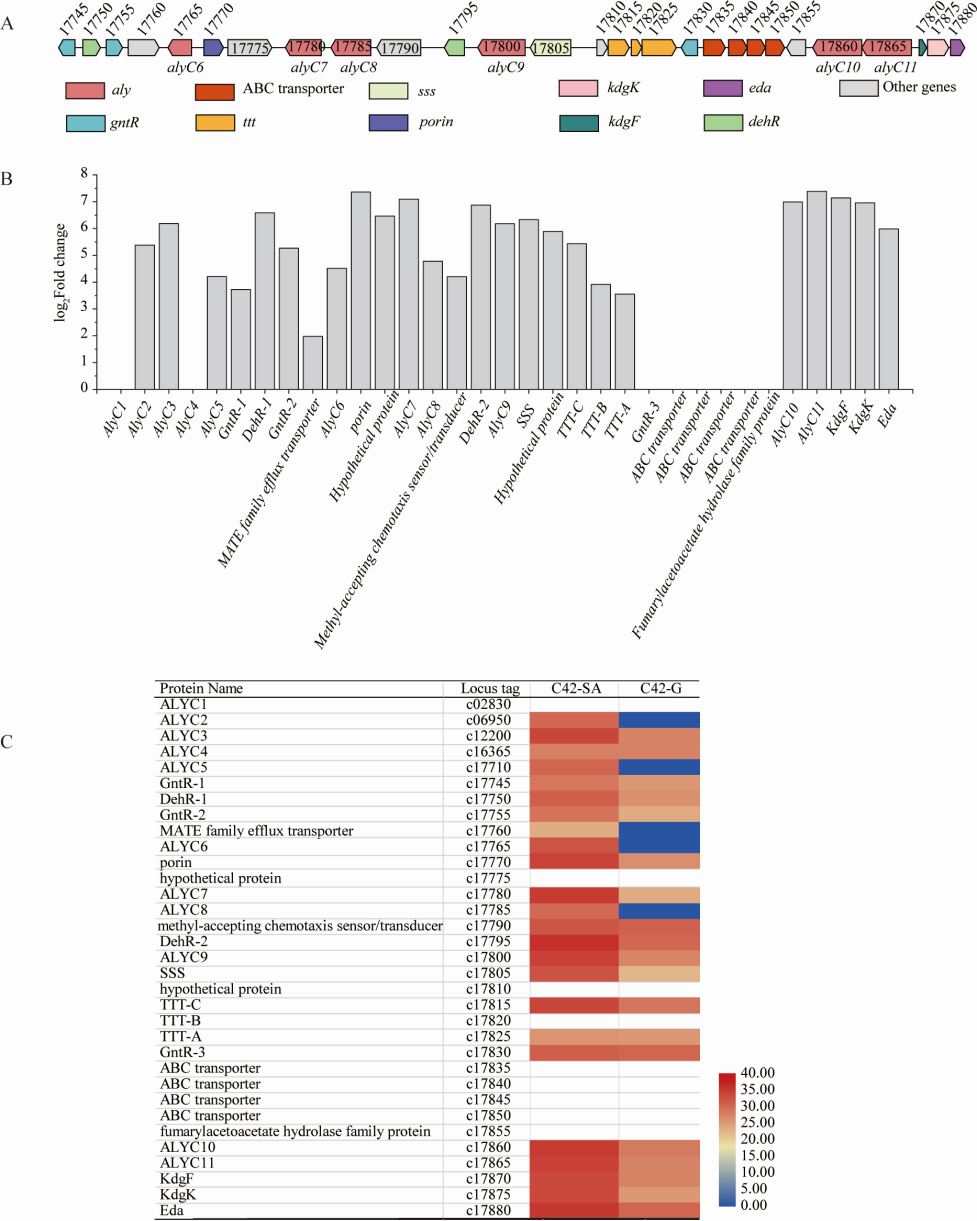
Identification of the alginate utilization locus in *Vibrio* sp. C42. (**A**) The alginate utilization locus in the genome of *Vibrio* sp. C42. *aly*, alginate lyase; *gntR*, GntR family transcriptional regulator; ABC transporter, ATP-binding cassette transporter; SSS, sodium-solute symporter; TTT, tripartite tricarboxylate transporter; *kdgK*, 2-dehydro-3-deoxy-gluconokinase; *kdgF*, pectin degradation protein; *eda*, 2-dehydro-3-deoxy-phosphogluconate aldolase; *dehR*, DEH reductase. (**B** and **C**) Comparative transcriptome (**B**) and proteome (**C**) analyses of *Vibrio* sp. C42 cells grown on SA and glucose. The dendrograms shown on the right of the heatmap in panel B depict the pairwise similarities between rows. Empty/white squares refer to genes that were not expressed or differentially expressed. Log_2_ fold change in panel C was log-transformed averages of RPKM values of genes expressed in cells grown on SA relative to glucose from duplicate experiments.

*Vibrio* sp. C42 was capable of utilizing sodium alginate (SA) as the sole carbon source for growth (Fig. S1), indicating that it could catabolize alginate for carbon and energy source. To investigate the mechanism of alginate utilization of this strain, the genome of *Vibrio* sp. C42 was sequenced and annotated (NCBI accession no. JAEKGD000000000.1). Based on gene function annotation, *Vibrio* sp. C42 contains 11 alginate lyase encoding genes (*alyC1–C11*). Among these, genes *alyC1*, *alyC2*, *alyC3*, *alyC4*, and *alyC5* are scattered in the genome, and the others (*alyC6–alyC11*) are in an AUL (Fig. 1A). The AUL of *Vibrio* sp. C42 contains 28 genes (~39.0 kbp). In addition to key enzymes related to alginate monosaccharide utilization, the six alginate lyase genes *alyC6-alyC11* (*JFJ08_17765*, *JFJ08_17780*, *JFJ08_17785*, *JFJ08_17800*, *JFJ08_17860*, and *JFJ08_17860*) are present. It contains five genes encoding enzymes involved in unsaturated alginate monosaccharide utilization (two *dehR* [*JFJ08_17750* and *JFJ08_17795*], *kdgF* [*JFJ08_17870*], *kdgK* [*JFJ08_17875*], and *eda* [*JFJ08_17880*]), three genes homologous to the transcriptional regulator gene *gntR* (*JFJ08_17745*, *JFJ08_17755,* and *JFJ08_17830*), nine genes encoding transport proteins (a porin [*JFJ08_17770*], a SSS [*JFJ08_17805*], a TTT [*JFJ08_17825*, *JFJ08_17820,* and *JFJ08_17815*], and an ABC transporter [*JFJ08_17835*, *JFJ08_17840*, *JFJ08_17845,* and *JFJ08_17850*]). The AUL also contains five genes that are not apparently related to alginate utilization, including genes encoding a MATE family efflux transporter (*JFJ08_17760*) (21), a methyl-accepting chemotaxis sensor/transducer (*JFJ08_17790*) (22), a fumarylacetoacetate hydrolase family protein (*JFJ08_17855*) (23), and two hypothetical proteins (*JFJ08_17775* and *JFJ08_17810*), whose functions are not investigated in this study.

To investigate whether the scattered alginate lyase genes and the genes in the AUL function in alginate utilization, transcriptome and proteome analyses were performed on *Vibrio* sp. C42 cultured with alginate as the sole carbon source. Among the five scattered alginate lyase genes, transcription of genes *alyC2*, *alyC3*, and *alyC5*, but not that of *alyC1* or *alyC4*, was upregulated in the alginate culture compared to those in the glucose culture (Fig. 1B). Transcription of the six alginate lyase genes (*alyC6–C11*) and five genes encoding enzymes (two *dehR*, *kdgF*, *kdgK*, and *eda*) involved in unsaturated alginate monosaccharide utilization in the AUL was upregulated (Fig. 1B). Among the genes encoding transport proteins, except the four genes encoding an ABC transporter, transcription of the others was all upregulated (Fig. 1B). Among the three genes encoding *gntR*, transcription of *gntR1* and *gntR2*, but not *gntR3*, was upregulated (Fig. 1B). Correspondingly, the genes with upregulated transcription all showed higher expression in the proteome in alginate culture than glucose culture, but those without upregulated transcription showed no detectable expression (*alyC1* and the ABC transporter genes) or no differential expression (*alyC4* and *gntR3*) between alginate and glucose culture (Fig. 1C). Noticeably, transcription of the three genes encoding the TTT was significantly upregulated in the transcriptome (Fig. 1B), although the membrane protein subunit TctB was not found in the proteome (Fig. 1C), possibly due to the instability of the membrane protein. Altogether, the transcription and expression of most of the scattered alginate lyase genes and the genes in the AUL were induced by alginate, suggesting that these genes are involved in alginate utilization in *Vibrio* sp. C42. Specifically, nine alginate lyases (AlyC2, AlyC3, AlyC5–AlyC11), key enzymes related to alginate monosaccharide utilization (DehR-1, DehR-2, KdgF, KdgK, and Eda), transport-related proteins (porin, SSS, and TTT), and two transcriptional regulatory proteins GntR-1 and GntR-2 may play roles in alginate utilization in *Vibrio* sp. C42. However, the alginate lyase AlyC1 and the ABC transporter, which did not show upregulation in the transcriptome, may not participate in alginate utilization under the tested conditions. AlyC1 may be regulated through pathways independent of this AUL. By contrast, the absence of upregulation of AlyC4 and the GntR-3 in the proteome suggests that they may be constitutively expressed. As AlyC4 is outside the AUL, it may serve a housekeeping role. Similar observations have been reported by Wong et al., who found that some alginate lyases were expressed constitutively and that the presence of alginate may not stimulate the expression of alginate lyases (1). Nevertheless, their precise roles in alginate utilization in *Vibrio* sp. C42 need further verification.

Although AULs have been reported in other bacteria (24), they were only recently reported in *Vibrio* by He et al. (7). They identified three AULs in *V. pelegius* WXL662. They also found that 21 alginate-degrading *Vibrio* genomes in the NCBI database have AULs. These AULs all contain genes encoding a variety of alginate-degrading enzymes, genes encoding transporters, including ABC transporter, porin, and SSS, and genes encoding regulators, including LysR, GntR, IclR, and KdgR (7). Similarly, the AUL of *Vibrio* sp. C42 contains genes encoding alginate-degrading enzymes, transporters (porin and SSS), and regulatory protein (GntR). In addition, we found that the AUL of *Vibrio* sp. C42 contains a TTT, which, to our knowledge, has not previously been reported to be involved in alginate utilization in bacteria.

### Extracellular degradation and intracellular catabolism of alginate by *Vibrio* sp. C42

**Fig 2:**
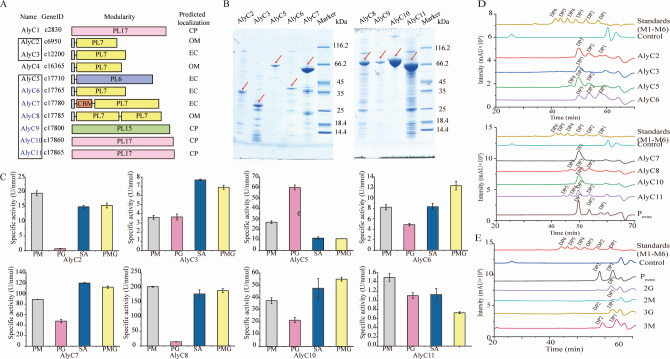
Enzymatic degradation of alginate by *Vibrio* sp. C42. (**A**) Putative alginate lyases in *Vibrio* sp. C42. Signal peptides are shown in gray box. CBM, carbohydrate-binding module; CP, cytoplasm; EC, extracellular space; OM, outer membrane. The alginate lyases in the AUL are shown in blue, those not in the AUL are in black, and those identified in the secretome are framed. The location of the alginate lyases which were not found in the secretome was predicted via Cello v2.5. (**B**) SDS-PAGE analysis of the purified AlyC2, AlyC3, and AlyC5–C11. (**C**) The substrate specificities of AlyC2, AlyC3, and AlyC5–C8, AlyC10, and AlyC11. The specific activities of the enzymes were measured toward PM, PG, SA, and PMG. Experiments were conducted in a 200-μL mixture containing 20 μL enzyme and 0.5 mg/mL substrate in 50 mM Tris-HCl (pH 8.0) and 0.5 M NaCl at 30°C for 10 min. The graphs show data from triplicate experiments (mean ± standard deviation). (**D**) HPLC analysis of the final degradation products of AlyC2, AlyC3, and AlyC5–C8, AlyC10, and AlyC11 toward SA. (**E**) HPLC analysis of the final degradation products of AlyC9 toward 2M, 2G, 3M, and 3G. Experiments were conducted in a 200 μL mixture containing 20 μL enzyme and 2 mg/mL SA in 50 mM Tris-HCl (pH 8.0) and 0.5 M NaCl at 30°C for 12 h. The products were analyzed by gel filtration chromatography using a Superdex peptide 10/300 GL column monitored at a wavelength of 210 nm. The control was treated with preheated inactivated lyases. Saturated mannuronate oligosaccharides from DP1 to DP6 were taken as the standards. DP, degree of polymerization.

The 11 alginate lyases of *Vibrio* sp. C42 are presented in Fig. 2. The figure summarizes their predicted protein modular architectures, as well as their substrate specificities and degradation products, determined experimentally. AlyC10 and AlyC11 belong to the PL17 family and have the highest sequence identity (99% and 95%) to the PL17 alginate lyases OalC (24) and OalB (24), respectively. AlyC2, AlyC3, AlyC4, AlyC6, AlyC7, and AlyC8 are all classified as the PL7 family enzymes, with the highest sequence identity (71%, 96%, 42%, 97%, 57%, and 90%) to the PL7 alginate lyases AlxM (25), AlyE (26), AlyA (26), AlyD (26), AlyDW11 (27), and AlyA (26), respectively. AlyC5 is a lyase of the PL6 family and has the highest sequence identity (93%) to the alginate lyase AlyF (28). AlyC9 belongs to the PL15 family, with the highest sequence identity (99%) to the alginate lyase OalA from *Vibrio splendidus* 12B01 (24). Coverage was 100% in every case. Among them, enzymes AlyC2–AlyC8 were predicted to possess a signal peptide, and the others did not (Fig. 2A).

To investigate the alginate lyases secreted by *Vibrio* sp. C42, secretome analysis was performed using liquid chromatography-tandem mass spectrometry (LC-MS/MS). Nine alginate lyases (AlyC2–C3 and AlyC5–C11) were detected in the secretome (Table 1). AlyC10 and AlyC11, which have no predicted signal peptide (Fig. 2A), showed relatively high abundance by mass spectrometry (MS) (Table 1) and may be secreted via other secretion systems (29). AlyC9 lacks a signal peptide (Fig. 2A), and its low abundance in the secretome (Table 1) suggests that it is likely an intracellular enzyme detected there, most probably as a result of cell lysis. Thus, *Vibrio* sp. C42 secreted at least eight alginate lyases, including AlyC2–C3, AlyC5–C8, and AlyC10–C11, for extracellular alginate degradation.

**TABLE 1:** Alginate lyases detected in the secretome of *Vibrio* sp. C42

Alginate lyase	Gene ID	Family	Coverage	Peptide	No. of PSMs^*a*^	Abundance^*b*^
AlyC8	c17785	PL7	40.03	16	32	0.33
AlyC7	c17780	PL7	33.52	10	24	0.25
AlyC3	c12200	PL7	24.06	7	11	0.12
AlyC10	c17860	PL17	16.18	10	11	0.12
AlyC11	c17865	PL17	13.12	7	8	0.08
AlyC6	c17765	PL7	21.74	4	5	0.05
AlyC5	c17710	PL6	5.02	2	2	0.02
AlyC9	c17800	PL15	3.03	2	2	0.02
AlyC2	c6950	PL7	4.21	1	1	0.01

^*a*^ No. of PSMs indicates the number of peptides that match the alginate lyase.

^*b*^ Abundance was calculated based on the proportion of the PSMs of an alginate lyase in the sum of PSMs of all alginate lyase in the secretome. The alginate lyases with an abundance value less than 5% in the secretome are not listed.

To investigate the extracellular degradation of alginate by *Vibrio* sp. C42, the nine predicted extracellular alginate lyases (AlyC2–C8 and AlyC10–C11) were expressed in *E. coli* and purified. AlyC1 was not pursued further in this study because it was not detected in the secretome or transcriptome analyses. As a result, except that the expression of AlyC4 failed, the other eight recombinant enzymes were successfully expressed and purified (Fig. 2B). The SDS-PAGE analysis showed anomalous migration of AlyC3 relative to its predicted molecular weight and differential migration positions of AlyC10 and AlyC11, despite their predicted similar molecular weights (Fig. 2B). LC-MS/MS peptide mapping confirmed their identities (Fig. S2), although the reason for these migration discrepancies remains unclear. In addition, *E. coli* BL21 harboring the empty vector showed no alginate degradation activity (data not shown), confirming that the observed activity is specifically attributed to the recombinant enzymes. These different enzymes were all capable of degrading four different alginate substrates, namely polymannuronate (PM), polyguluronate (PG), the alternating copolymer poly-mannuronate-guluronate (PMG), and SA, but with different substrate specificities (Fig. 2C). AlyC5 showed the highest activity toward PG (1,019 ± 39 U/mg). AlyC2 and AlyC8 showed the highest activity toward PM (605 ± 26 U/mg and 3,084 ± 24 U/mg, respectively). AlyC6 and AlyC10 showed the highest activity toward PMG (322 ± 22 U/mg and 683 ± 19 U/mg, respectively). AlyC3 and AlyC7 showed the highest activity toward SA (201 ± 2 U/mg and 2,102 ± 16 U/mg, respectively). The complementary substrate selectivity of these enzymes enables *Vibrio* sp. C42 to cleave various bonds in alginate, which is conducive to the complete degradation of alginate.

When SA was degraded, the final degradation products of AlyC3, AlyC7, and AlyC10 were solely trimers, while those of AlyC2, AlyC5, AlyC6, AlyC8, and AlyC11 were mainly trimers (Fig. 2D). These results indicate that all the secreted enzymes are endo-alginate lyases. In addition, when SA was degraded by a mixture of all the secreted alginate lyases (AlyC2–C3, AlyC5–C8, and AlyC10–C11), the final degradation products (*P*_extra_) were mainly trimers and a small proportion of dimers and unsaturated monomers (Fig. 2D).

To investigate the intracellular degradation of alginate oligosaccharides by *Vibrio* sp. C42, the predicted intracellular alginate lyase AlyC9 was expressed in *E. coli* and purified (Fig. 2B). Because the oligosaccharides in the *P*_extra_ generated by the complex of extracellular alginate lyases on alginate were mostly di- and trisaccharides, the activities of AlyC9 toward di- and trisaccharides were detected using commercially obtained saturated oligosaccharides (2M, 3M, 2G, 3G) as the substrates (Fig. 2E). The results showed that AlyC9 could degrade all these di- and trisaccharides into monosaccharides, confirming that AlyC9 is an intracellular alginate oligosaccharide lyase.

Among the putative enzymes involved in the utilization of unsaturated alginate monosaccharides in the AUL, KdgF shares 67% sequence identity (100% coverage) with KdgF from *Yersinia enterocolitica* (9), DehR-1 shares 71% sequence identity (100% coverage) with A1-R′ from *Sphingomonas sp*. A1 (30), DehR-2 shares 34% sequence identity (100% coverage) with GarR from *Salmonella typhimurium* (31), KdgK shares 54% sequence identity (100% coverage) with YdjH from *Acinetobacter baumannii* (32), and Eda shares 42% sequence identity (100% coverage) with KDPG aldolase from *E. coli* (33). Thus, it is most likely that the unsaturated alginate monosaccharides in cells of *Vibrio* sp. C42 are finally converted to G3P by KdgF, DehR, KdgK, and Eda, as in other bacteria (5). To confirm this, these enzymes (KdgF, DehR-1, DehR-2, KdgK, and Eda) were expressed in *E. coli* and purified, and their activities were detected. As a result, KdgF activity was observed by monitoring the decrease in *A*_235_ after the addition of KdgF to the monosaccharides produced by AlyC9 (∆*A*_235_ = −0.73 ± 0.04). The activities of DehR-1 and DehR-2 were observed by monitoring the oxidation of NADH (the decrease at the *A*_340_) in the presence of DEH (∆*A*_340_ = −0.62 ± 0.01 and ∆*A*_340_ = −0.74 ± 0.06, respectively). The activities of KdgK and Eda were observed by monitoring the oxidation of NADH in the presence of KDG (∆*A*_340_ = −0.96 ± 0.06) and KDPG (∆*A*_340_ = −0.12 ± 0.04), respectively. These results indicated that, in the alginate utilization process of *Vibrio* sp. C42, the intracellular alginate monosaccharides were catabolized by KdgF, DehR-1, DehR-2, KdgK, and Eda.

While many studies focused on the biochemical properties of individual alginate lyases from *Vibrio*, there are only a few reports on the enzymatic mechanism of alginate degradation and utilization of *Vibrio*. Zhang et al. summarized strategies for alginate utilization of *Vibrio*. Poly-alginate lyases initiate extracellular degradation, and the resulting oligosaccharides are further processed in the periplasm and intracellularly by oligo-alginate lyases (12). He et al. investigated 11 alginate lyases in *V. pelegius*. VpAly-I, II, and IV–VII (belonging to families PL7 and PL6) degrade alginate (SA, PM, and PG) into DP3–6 extracellularly; VpAly-III (belonging to family PL7) degrades DP4–6 to DP3 in the periplasmic space; and VpAly-VIII–XI (belonging to families PL17 and PL14) degrade DP3 into monosaccharides intracellularly (7). In *Vibrio* sp. B1Z05, alginate lyases VBAly5, VBAly6, VBAly7, VBAly16, and VBAly17 (belonging to families PL7 and PL6) degrade alginate (SA, PM, and PG) into DP2–6 extracellularly (14). VBAly10 and VBAly11 (belonging to family PL17) (14) and VBAly15A (belonging to family PL15) (15) degrade these oligosaccharides into monosaccharides intracellularly. In *Vibrio* sp. C42, alginate lyases are distributed exclusively within the cytoplasm and extracellular space, with no alginate lyases predicted in the periplasmic space. The extracellular alginate lyases (belonging to PL6, PL7, and PL17 families) degrade alginate (SA, PM, PG, and PMG) mainly into DP3. Research on the subsequent utilization mechanisms of alginate monosaccharides is limited and primarily focused on *Bacillus* (5). Here, we demonstrated the functions of KdgF, DehR-1, DehR-2, KdgK, and Eda, thereby fully elucidating the enzymatic mechanism for alginate degradation and utilization in *Vibrio* sp. C42.

### Both SSS and TTT are involved in alginate oligosaccharide uptake by *Vibrio* sp. C42

The AUL of *Vibrio* sp. C42 encodes four transporters, including a porin, a SSS, a TTT, and an ABC transporter. Because the above transcriptome and proteome data suggested that the ABC transporter may not function in alginate utilization in *Vibrio* sp. C42, the ABC transporter was not further investigated in this study. The porin shares 96% sequence identity (100% coverage) to the porin (KdgM) from *V. splendidus*, which is responsible for the transport of alginate oligosaccharides into the periplasmic space (8). The SSS shares the 63% sequence identity (100% coverage) to the SSS (ToaABC) from *V. splendidus* (8). For the three components of the TTT, TTT-A shares 37% sequence identity (46% coverage) to the TctA_ar from a metagenomic library of activated sludge (34), and TTT-C shares 31% sequence identity (88% coverage) with BugD from *Bordetella pertussis* (18). To date, no TctB from TTT has been characterized. Therefore, no reliable reference sequence was available for sequence identity comparison. In addition, repeated attempts to purify TctB were unsuccessful, preventing further biochemical characterization. Consequently, TctB was excluded from the sequence comparison and further functional analysis.

**Fig 3:**
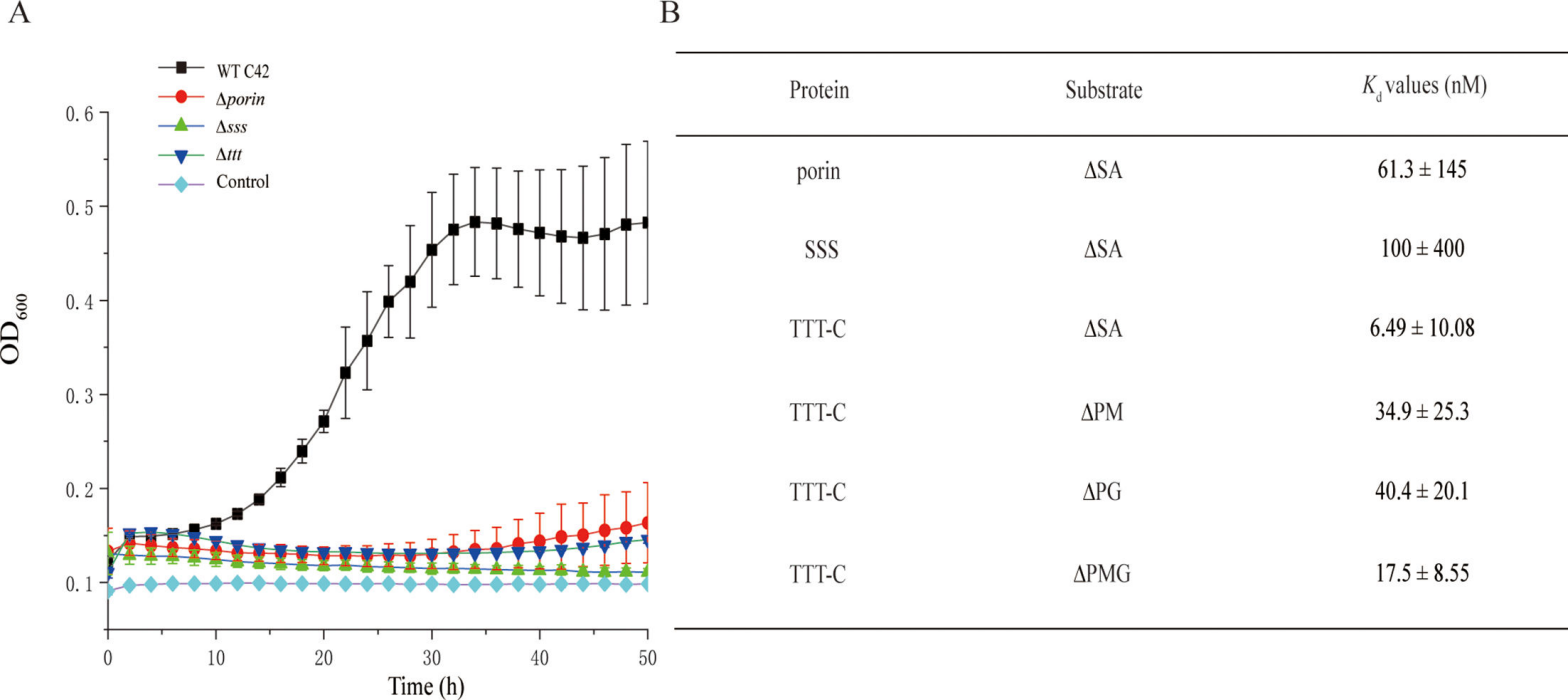
Functional analyses of the transporters. (**A**) Growth curves of wild-type *Vibrio* sp. C42 and the gene deletion mutants *Δporin*, *Δsss,* and *Δttt* cultivated with alginate as the sole carbon source. The culture without a carbon source was treated as the control. (**B**) Binding capacities of porin, SSS, and TTT-C to unsaturated alginate oligosaccharides. Data are from triplicate experiments (mean ± standard deviation).

To investigate the roles of the porin, SSS, and TTT in alginate oligosaccharide uptake by *Vibrio* sp. C42, we performed gene knockouts and constructed three single-deletion mutants: *Δporin*, *Δsss*, and *Δttt*. Growth observation showed that gene deletion of the mutants all severely affected their growth on SA (Fig. 3A), indicating that all three transporters were essential for the uptake of alginate oligosaccharides in *Vibrio* sp. C42. To further confirm the roles of the porin, SSS, and TTT proteins in alginate oligosaccharide transport, we sought to reconstitute the ligand-binding step. For this purpose, the substrate-binding proteins were expressed in *E. coli*, and the ability of the recombinant proteins to bind ∆SA (the degradation products of SA by the mixture of the secreted alginate lyases) was detected using ITC (Fig. 3B; Fig, S3A). The results showed that all of them had ∆SA-binding ability, with *K*_*d*_ (dissociation constant) values of 6 ± 11 nM (porin), 100 ± 400 nM (SSS), and 61 ± 145 nM (TTT-C, the substrate-binding protein of the TTT), respectively.

Since TTT was first found to be involved in bacterial uptake of alginate oligosaccharides, we further analyzed the substrate affinity of TTT-C. In addition to ∆SA, TTT-C exhibited high binding ability to unsaturated alginate oligosaccharides ∆PM, ∆PG, and ∆PMG (degradation products of PM, PG, and PMG by the complex of the secreted alginate lyases, respectively), with *K*_*d*_ values of 35 ± 25 nM (∆PM), 40 ± 20 µM (∆PG), and 18 ± 9 nM (∆PMG), respectively (Fig. 3B; Fig. S3B), indicating that TTT-C can bind various alginate oligosaccharides. TTT-C exhibits low similarity (<35%) to previously reported TTT substrate-binding proteins (SBPs). However, ITC measurement showed that TTT-C did not bind the reported substrates of TTT SBPs, including citrate, D-glucuronic acid, and DL-malic acid (16). Thus, TTT-C is specific to unsaturated alginate oligosaccharides.

Currently, bacterial transporters for alginate degradation products are primarily categorized into three types: Pit-ABC transport system in *Sphingomonas* (3), SusC/SusD-MFS transport system in *Flavobacteriaceae* (8), and porin-SSS system in *Vibrio* (7). However, among these, the porin-SSS system in the alginate utilization pathway in *Vibrio* has not been experimentally validated. Here, our results provide the first experimental evidence for the porin-SSS system in alginate utilization in *Vibrio* sp. C42. Specifically, the outer membrane porin transports extracellular oligosaccharides (primarily di- and trisaccharides) into the periplasm, and the inner membrane SSS transporter mediates their translocation from the periplasm into the cytoplasm. Furthermore, our results demonstrated that, in addition to the porin-SSS system, a TTT on the inner membrane is also involved in the transport of alginate oligosaccharides in *Vibrio* sp. C42, which represents the first report of a TTT protein specific to unsaturated alginate oligosaccharides.

### Structural insight into the binding specificity of TTT-C to unsaturated alginate oligosaccharides

**Fig 4:**
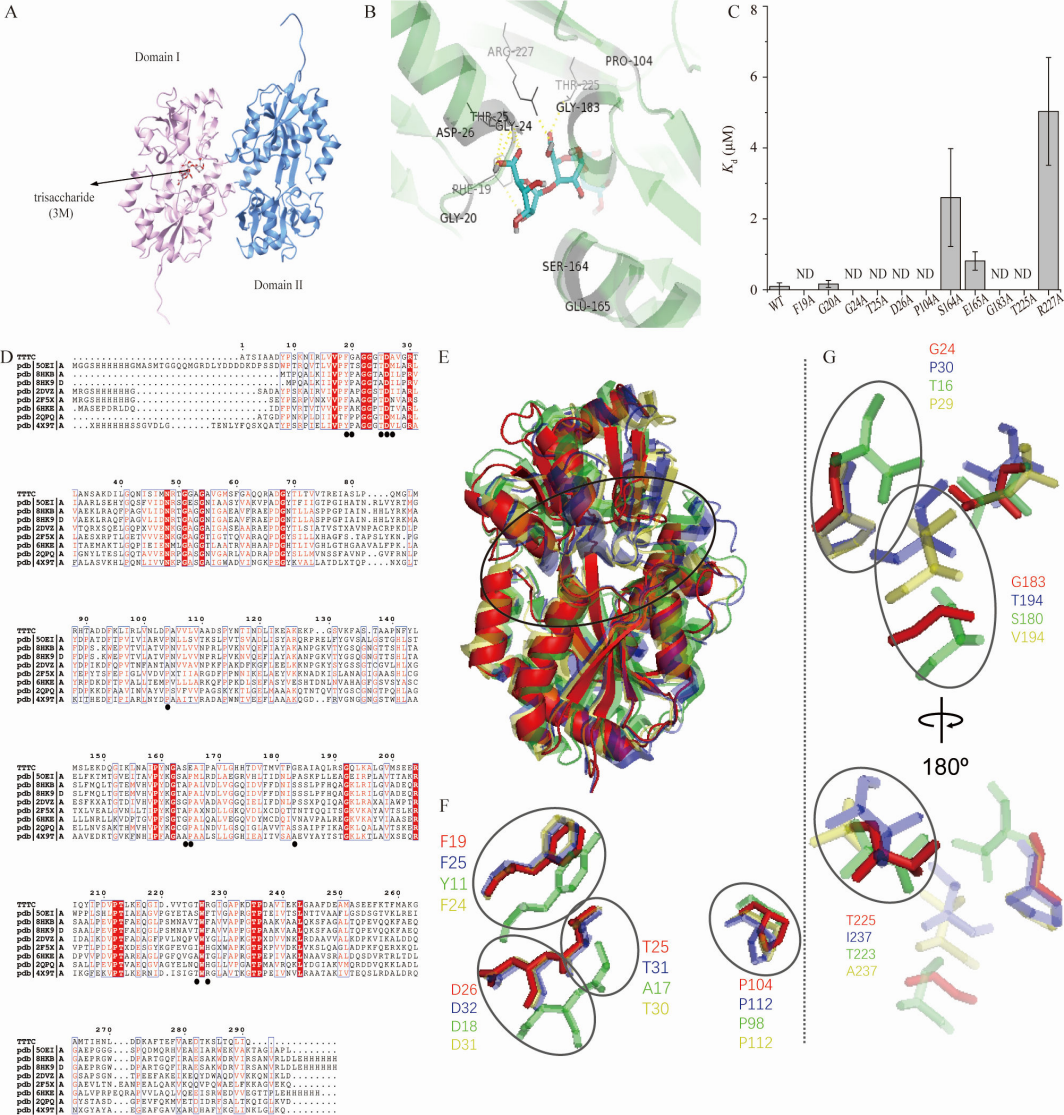
Characterization of TTT-C. (**A**) Overall structure of TTT-C/3M. Domain I is colored in pink, and domain II blue. The trisaccharide molecule is shown as cyan sticks. (**B**) Key residues of TTT-C interacting with trisaccharide. (**C**) Binding abilities of TTT-C mutants to ∆SA. *K*_*d*_ values were determined by ITC in Tris-HCl (10 mM; pH 8.0) containing 100 mM NaCl. The concentration of ∆SA was 200 µM. The concentration of proteins was 20 µM. Experiments were performed in triplicate. ND, not binding detected. (**D**) Multiple sequence alignment of TTT-C and other TTT SBPs. (**E**) Structural alignment of TTT-C with BugD, IsTBP, and MatC. The binding pockets are circled. (**F **and **G**) Structural alignment of the conserved residues (**F**) and non-conserved residues (**G**) among TTT-C, BugD, IsTBP, and MatC. TTT-C is shown in red, BugD in blue, IsTBP in green, and MatC in yellow. Key residues are shown as sticks.

To further reveal the mechanism by which TTT-C specifically binds unsaturated alginate oligosaccharides, the crystal structure of TTT-C was solved for the first time (Fig. 4A). TTT-C comprises two domains (domains I and II) (Fig. 4A), similar to other TTT SBPs. Structure-based homology searches of TTT-C in the PDB database revealed that the closest structural homolog of TTT-C is BugD (PDB: 2F5X; RMSD: 1.72 Å; Z-score: 31), a citrate-binding SBP (18).

Potential binding sites for an alginate trisaccharide (3M, GlyTouCan: G78314GJ) in TTT-C were predicted using the CB-DOCK2 web server (http://clab.labshare.cn/cb-dock2/). The Vina Score value is −8.9 kcal/mol, which is below the −7.0 kcal/mol threshold, suggesting a favorable and reliable binding mode. Based on the structural analysis, 11 amino acid residues (Phe^19^, Gly^20^, Gly^24^, Thr^25^, Asp^26^, Pro^104^, Ser^164^, Glu^165^, Gly^183^, Thr^225^, and Arg^227^) in TTT-C were predicted to interact with the alginate trisaccharide substrate (Fig. 4B). To further identify the key residues involved in the binding of alginate oligosaccharides, mutation of each residue to alanine was performed. ITC analysis indicated that the binding affinities of mutants F19A, G24A, T25A, D26A, P104A, G183A, and T225A to ∆SA were completely abolished and the *K*_*d*_ values of G20A (16 ± 10 µM), S164A (260 ± 138 µM), E165A (81 ± 26 µM), and R227A (513 ± 52 µM) were decreased compared to the wild-type TTT-C (Fig. 4C; Fig. S4). These results indicate that amino acid residues Phe^19^, Gly^24^, Thr^25^, Asp^26^, Pro^104^, Gly^183^, and Thr^225^ in TTT-C are essential for alginate oligosaccharide binding, while residues Gly^20^, Ser^164^, Glu^165^, and Arg^227^ also play a role in the binding process.

Based on multiple sequence alignment of TTT-C with other characterized TTT SBPs, the key amino acid residues Phe^19^, Thr^25^, Asp^26^, and Pro^104^ are conserved among them (Fig. 4D). However, the key residues Gly^24^, Gly^183^, and Thr^225^ of TTT-C exhibit differences from the corresponding residues reported in other TTT SBPs, such as BugD (18), IsTBP (35) (PDB: 8HK9 and 8HKB), MatC (36) (PDB: 6HKE), and Rpa4515 (37) (PDB: 5EOI).

To further elucidate these differences, structural alignment was conducted. Due to substantial structural divergence, Rpa4515 was excluded, and comparisons were limited to TTT-C with BugD (18), IsTBP (35), and MatC (36) (Fig. 4E through G). Overall, these proteins share a similar fold; however, TTT-C displays a noticeably larger binding pocket (Fig. 4E). Among the conserved residues, Phe^19^, Thr^25^, Asp^26^, and Pro^104^ adopt similar conformations to their counterparts in BugD and MatC and show slight positional shifts compared to those in IsTBP (Fig. 4F). In contrast, the non-conserved residues exhibit more pronounced differences. Specifically, Gly^24^ displays both a positional shift and a reduced side chain, Gly^183^ shows a positional displacement, and Thr^225^ has a shorter side chain compared to the corresponding residues in BugD and MatC (Fig. 4G). These deviations are even more pronounced when compared with IsTBP. Functionally, the corresponding residues in other SBPs are known to interact with distinct substrates. For example, Pro^29^ in BugD and Pro^30^ in MatC (corresponding to Gly^24^ in TTT-C) are involved in binding the carbon backbone of citrate. Ser^207^ in IsTBP (corresponding to Gly^183^ in TTT-C) participates in terephthalate binding, while Ser^261^ in Rpa4515 (corresponding to Thr^225^ in TTT-C) coordinates the distal carboxylate group of 2-oxoadipate via hydrogen bonding. Collectively, these structural variations in TTT-C result in an expanded binding cavity, which is likely adapted to accommodate the larger unsaturated alginate trisaccharide. The non-conserved residues, particularly Gly^24^, Gly^183^, and Thr^225^, are therefore proposed to play critical roles in substrate specificity, distinguishing TTT-C from other TTT SBPs that recognize structurally distinct ligands.

TTT transporters constitute the most abundant protein family in β-Proteobacteria (38). In recent years, the biochemical properties and structures of TTTs have been reported. TTTs can transport fluorocitrate, citrate, isocitrate, cis-aconitate (16), aldarate, and D-glucarate (19). Compared to other TTT SBPs, although TTT-C of *Vibrio* sp. C42 has a similar overall structure, several key residues (Gly^24^, Gly^183^, and Thr^225^) involved in substrate binding have changed, which likely results in the specificity of TTT-C to unsaturated alginate oligosaccharides.

### The alginate utilization pathway of *Vibrio* sp. C42 and the universality of alginate utilization loci containing TTT genes in marine bacteria

**Fig 5:**
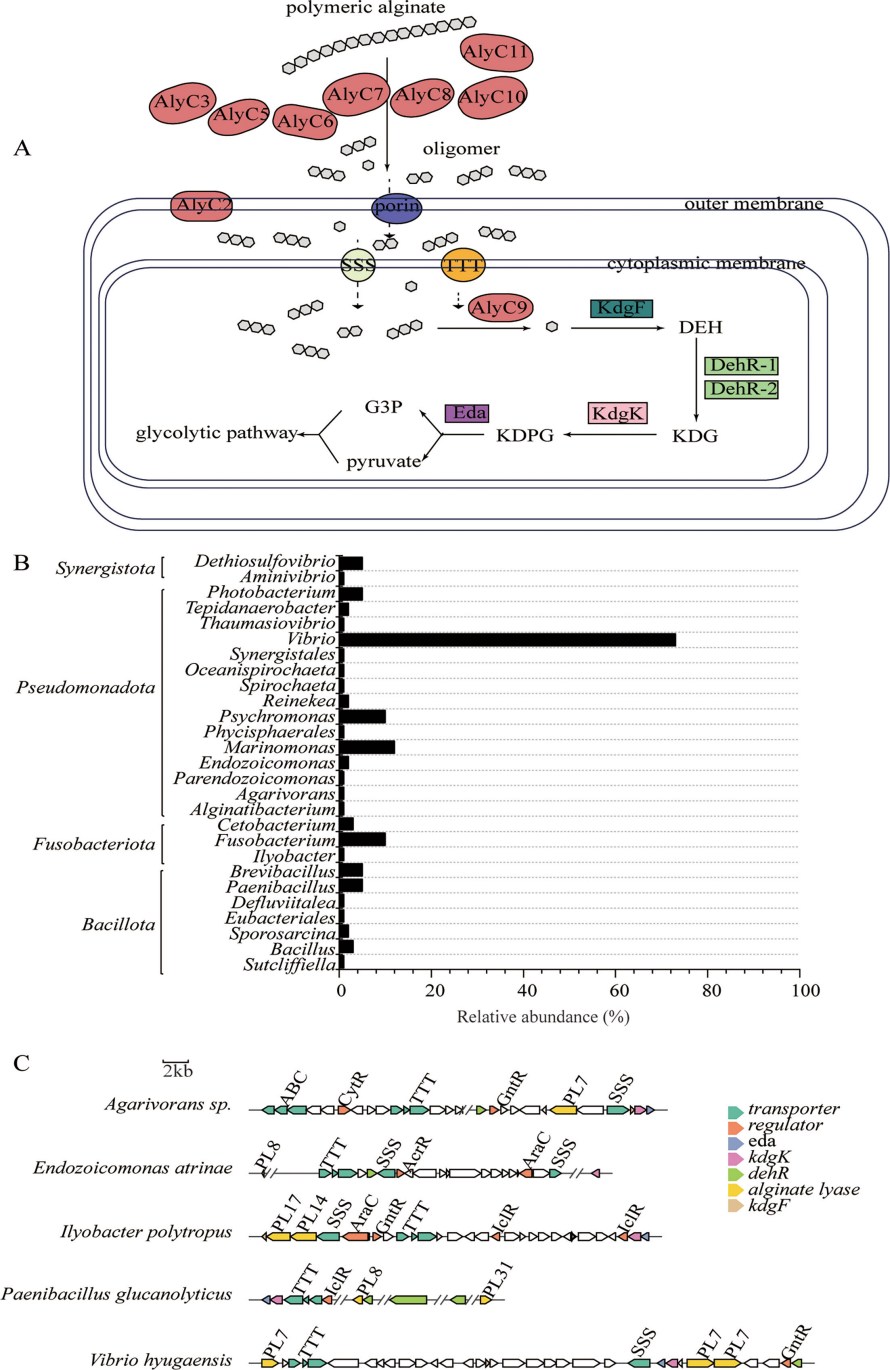
Distribution of TTT-containing AULs in bacteria. (**A**) Schematic diagram of the alginate utilization pathway in *Vibrio* sp. C42. Eight extracellular alginate lyases degrade alginate into oligomers and monomers. These saccharide products are transported into the periplasm by the porin and then into the cytoplasm by SSS and TTT. Oligomers are decomposed into unsaturated monomers by alginate lyase AlyC9 in the cytoplasm. Unsaturated monomers are stepwise converted to glyceraldehyde-3-phosphate (G3P) by KdgF, DehR, KdgK, and Eda. DEH, 4-deoxy-L-*erythro*-5-hexoseulose uronic acid; KDG, 2-keto-3-deoxy-D-gluconate; KDPG, 2-keto-3-deoxy-6-phosphogluconate. (**B**) Diversity of bacteria containing TTT-C homologs. (**C**) Diversity of TTT-containing AULs in bacterial genomes. GntR, GntR family transcriptional regulator; CytR, CytR family transcriptional regulator; AcrR, AcrR family transcriptional regulator; AraC, AraC family transcriptional regulator; IclR, IclR family transcriptional regulator; ABC, ATP-binding cassette transporter; TTT, tripartite tricarboxylate transporter; kdgK, 2-dehydro-3-deoxy-gluconokinase; kdgF, pectin degradation protein; eda, 2-dehydro-3-deoxy-phosphogluconate aldolase; dehR, DEH reductase; Na, sodium-solute symporter.

Based on the above data, we propose the following alginate utilization pathway in *Vibrio* sp. C42. As shown in Fig. 5A, at least eight extracellular alginate lyases (AlyC2, AlyC3, AlyC5-AlyC8, AlyC10, and AlyC11) degrade alginate mainly into trimers, with a small portion of dimers and monomers. The produced saccharides are transported into the periplasmic space by the porin across the outer membrane and then transported into the cytoplasm by the SSS and the TTT. Intracellular alginate oligosaccharides are further decomposed into unsaturated monomers by the alginate lyase AlyC9 in the cytoplasm. The unsaturated monomers are finally converted to G3P by KdgF, DehR, KdgK, and Eda step by step, which then enter the glycolytic pathway for further utilization (Fig. 5), similar to the intracellular utilization of unsaturated monomers in other alginate-degrading bacteria (5).

To investigate the prevalence of TTT in the alginate utilization pathway of marine bacteria, the distribution of TTT in bacterial genomes was investigated with TTT-C as a query sequence in IMG database (https://img.jgi.doe.gov). As a result, a total of 154 homologs (E value <1e−50, identity >45%) were obtained. The retrieved sequences were predominantly distributed in phyla Bacteroidetes (18/152), Fusobacteria (14/152), Proteobacteria (114/152), and Proteobacteria (6/152), belonging to 17 families and 27 genera (Fig. 5B). Among these, Vibrionaceae was the dominant family (81/152), and *Vibrio* was the dominant genus (73/152). These data indicate that the TTT-C homologs are widely distributed across bacterial groups and primarily concentrated within *Vibrio*.

After further manual curation of each genome, only 29 of the 154 TTT-C-harboring strains were found to have a TTT-containing AUL. These strains mostly belong to *Vibrio* (73%), with a minority in genera *Agarivorans* (3%), *Endozoicomonas* (7%), *Ilyobacter* (3%), and *Paenibacillus* (14%). These AULs contain genes encoding all key proteins for alginate utilization, including enzymes, transporters, and regulatory proteins (Fig. 5C), suggesting that these TTT-containing AULs are responsible for alginate utilization in the bacterial strains.

To determine whether the TTT-C homologs have the same substrate specificity as TTT-C, we randomly selected one sequence close to the minimum threshold (TTT-PG, NCBI accession no. WP_036641827.1, with query sequence similarity of 43%) and one sequence close to the minimum threshold in *Vibrio* (TTT-VR, NCBI accession no. WP_040992967.1, with query sequence similarity of 84%) from the 29 screened strains for heterologous expression. ITC analysis indicated that both proteins had ∆SA-binding ability, with the *K*_*d*_ values of 225 ± 173 µM (TTT-PG) and 412 ± 200 µM (TTT-VR), respectively (Fig. S5).

Previous studies have demonstrated bacterial pathways for alginate utilization (12), highlighting the significant role of bacterial alginate metabolism in the marine carbon cycle. However, TTT has not been reported in bacterial AULs. Here, we elucidated the mechanism of alginate utilization of *Vibrio* sp. C42 and identified the role of TTT in alginate oligosaccharide transport. Bioinformatic analysis further revealed that AULs containing TTT are widely distributed among diverse marine bacteria, suggesting their important involvement in marine alginate degradation and cycling.

Collectively, this study expands the current understanding of bacterial alginate utilization by revealing the involvement of a previously unrecognized TTT system in the uptake of unsaturated alginate oligosaccharides. Previous studies on alginate utilization pathways in *Vibrio* mainly focused on alginate-degrading enzymes and the proposed porin-SSS transport system, which has not yet been experimentally validated. In contrast, our results demonstrate that *Vibrio* sp. C42 employs a more complex transport strategy in which both SSS and TTT participate in alginate oligosaccharide uptake. Structural and mutational analyses further revealed that TTT-C has evolved a distinct substrate-binding pocket adapted for binding unsaturated alginate oligosaccharides, distinguishing it from previously characterized TTT substrate-binding proteins that recognize small organic acids or aromatic compounds. Distribution analysis of TTT-containing AULs in marine bacteria further suggests that this transport strategy may represent an important but previously overlooked component of marine alginate metabolism. Given the ecological importance of alginate as one of the major polysaccharides in brown algae, the identification of this novel TTT-mediated transport pathway provides new insights into the diversity of marine polysaccharide utilization systems and contributes to a deeper understanding of bacterial participation in marine carbon cycling.

### Conclusion

In this study, we analyzed the complete alginate utilization pathway in *Vibrio* sp. C42, encompassing enzyme systems and transport systems. This pathway is generally consistent with the previously reported AUL system. However, a novel TTT, which is specific to alginate oligosaccharides and involved in alginate oligosaccharide transport, was identified in this pathway. Bioinformatic analysis revealed that this TTT-containing alginate utilization pathway is widely distributed in marine bacteria, mostly in *Vibrio*, suggesting their importance in marine alginate metabolism. Therefore, this study reveals a new alginate utilization pathway containing TTT in marine bacteria, shedding light on marine alginate degradation and cycling.

## MATERIALS AND METHODS

### Materials and strains

Alginate lyase-excreting *Vibrio* sp. C42 was isolated from a *Sargassum* sample collected from the coastal seawater (37°9′24.7″N, 122°35′35.9″E) in Shandong Province, China, and cultured in lysogeny broth (LB) medium. The chemicals and reagents used in this study are of analytical grade. SA (purity >98.0%) derived from brown algae was purchased from Sigma (America). Polymannuronate (PM, 6–8 kDa, purity >95%) and polyguluronate (PG, 6–8 kDa, purity >95%) were purchased from Zstandard (China). Oligosaccharide substrates (1–6M and 1–6G) with purity >98% were purchased from BZ Oligo Biotech Co., Ltd. (China). PMG was prepared as previously described (39). *Escherichia coli* strains DH5α and BL21 (DE3) were from Tsingke (China) and cultured in LB medium. *E. coli* strain WM3064 was preserved in our laboratory.

### Cultivation and growth detection

*Vibrio* sp. C42 was cultured in 200 μL minimal medium (0.05% [wt/vol] NH_4_Cl, 3% [wt/vol] NaCl, 0.3% [wt/vol] MgCl_2_·6H_2_O, 0.2% [wt/vol] K_2_SO_4_, 0.02% [wt/vol] K_2_HPO_4_, 0.001% [wt/vol] CaCl_2_, 0.0006% [wt/vol] FeCl_3_·6H_2_O, 0.0005% [wt/vol] Na_2_MoO_4_·7H_2_O, 0.0004% [wt/vol] CuCl_2_·2H_2_O, and 0.6% [wt/vol] Tris [pH 7.5–8.0]) containing 0.5% alginate or 0.2% (wt/vol) glucose at 25°C. The growth curves of *Vibrio* sp. C42 were generated by measuring the optical density at 600 nm (OD_600_) of the cultures using a spectrophotometer (V-550, Jasco Corporation, Japan). The culture without a carbon source was treated as the control.

### Sequencing of the genomic DNA

The genomic DNA of *Vibrio* sp. C42 was extracted using a BioTeke DNA extraction kit (China), and sequenced on the Illumina Hiseq sequencing platform (Majorbio, China). The genome assembly was performed using the ABySS 2.1.5 analysis process implemented in SMRT Link (V6.0.0.47841) to get the draft genome (40).

### Phylogenetic analysis, gene function annotation, and protein localization prediction

Gene functions were annotated using the RAST server (http://rast.nmpdr.org/) (41, 42). Information on the classification of CAZymes was derived from the CAZy database (http://www.cazy.org/) (43). Conserved protein modules of each protein were queried by BlastP searches against the GenBank non-redundant database. Signal peptides were predicted using SignalP v5.0 (http://www.cbs.dtu.dk/services/SignalP/). Cello v2.5 was used to predict the localization of proteins (44).

### Secretome analysis

*Vibrio* sp. C42 was cultured in 80 mL minimal medium containing 0.5% alginate at 25°C to the mid-log phase (OD_600_ ≈0.2). Then, the culture was centrifuged, and the supernatant was collected. The proteins in the supernatant were precipitated by 50 mL acetone solution containing 10% trichloroacetic acid and 0.1% dithiothreitol overnight at −20°C. The protein pellets were collected by centrifugation, washed twice with 80% acetone solution and once with 100% acetone solution, and then dried. The dried proteins were completely digested with trypsin, and the resultant peptides were subjected to LC-MS/MS analysis at the Institute of Genetics and Development Biology, Chinese Academy of Sciences, with reference to Li et al. (45). Finally, the data were analyzed by Thermo Scientific Proteome Discoverer software version 1.4.

### Transcriptome analysis

*Vibrio* sp. C42 was cultured in duplicate in 50 mL of minimal medium containing 0.2% (wt/vol) glucose or 0.2% (wt/vol) alginate at 25°C to the mid-log phase. There were two repeats for each treatment. Cells were collected by centrifugation at 4°C, 4000 × *g* for 5 min. Then, the total RNA of the cells was extracted using TRIzol Reagent (Invitrogen, USA), and RNA-seq strand-specific libraries were prepared using 5 μg of RNA extracted from each sample with the TruSeq RNA sample preparation kit (Illumina, USA) and the RiboZero rRNA removal kit (Epicenter, USA). High-throughput sequencing was performed in paired-end reads on the Illumina Hiseq 6000 platform, and high-quality clean reads were aligned to the genome of *Vibrio* sp. C42 using Rockhopper. The differential gene expression analysis was performed by EdgeR (46), and transcript levels were compared using the reads per kilobase per million mapped reads (RPKM) method (47). Furthermore, fold change ≥2 and false discovery rate (FDR) <0.01 were used as criteria to screen differentially expressed genes.

### Proteome analysis

The cells of *Vibrio* sp. C42 were prepared as described in “Transcriptome analysis,” above, and sent to Shanghai Applied Protein Technology (China) for proteome analysis. Briefly, the samples were lysed with SDT buffer (4% SDS, 1 mM DL-Dithiothreitol [DTT], 100 mM Tris-HCl, pH 7.6) to extract proteins. After quantification with a Pierce bicinchoninic acid (BCA) protein assay kit (Thermo, USA), 200 μg of proteins from each sample was digested according to the filter-aided sample preparation procedure (FASP) (48). The resulting peptides were analyzed by MS and MS/MS using MaxQuant (49). After pairwise correction of the peptide and protein multilayers, label-free quantification (LFQ) values were derived (50). The LFQ values were then averaged for each protein.

### Gene cloning, protein expression, and purification

Genes encoding AlyC2–C11, DehR-1, DehR-2, KdgK, KdgF, Eda, porin, SSS, TTT-C, TTT-PG, TTT-VR, GntR-1, and GntR-2 were amplified from the genomic DNA of *Vibrio* sp. C42 via PCR and cloned into the vector pET-22b between the restriction sites *NdeI* and *XhoI* along with a C-terminal His-tag. For soluble proteins (AlyC2–C11, DehR-1, DehR-2, KdgK, KdgF, Eda, TTT-C, TTT-PG, TTT-VR, GntR-1, and GntR-2), recombinant proteins were overexpressed in *E. coli* BL21 (DE3). For membrane proteins (porin and SSS), the expression host was *E. coli* C43 (DE3). Cells were grown at 37°C and 180 rpm in LB broth containing 100 μg/mL ampicillin. When the OD_600_ of the culture reached 0.6, the expression of the recombinant proteins was induced by 0.3 mM isopropyl-β-D-thiogalactopyranoside (IPTG) at 15°C and 100 rpm for 16 h. After cultivation, the cells were harvested by centrifugation at 4°C, 4,000 × *g* for 10 min, and disrupted using a JN-02C French press (JNBIO, China) in the buffer containing 50 mM Tris-HCl (pH 8.0) and 100 mM NaCl. The resultant mixtures were centrifuged at 4°C, 12,000 × *g* for 60 min, and the supernatants were collected. For membrane proteins (porin and SSS), the supernatant was further subjected to ultracentrifugation at 150,000 × *g* for 60 min at 4°C to collect the membrane pellet. The pellet was resuspended in solubilization buffer (50 mM Tris-HCl, 200 mM NaCl, 5% glycerol, 0.1 mM phenylmethylsulfonyl fluoride, 0.1 mM DL-dithiothreitol, 1% [wt/vol] n-dodecyl-β-D-maltoside [DDM], pH 8.0) and stirred overnight at 4°C. The solubilized mixture was ultracentrifuged again at 150,000 × *g* for 30 min at 4°C, and the supernatant was collected. The target proteins were then purified by NTA-Ni Sepharose affinity chromatography (Qiagen, Germany). The resultant protein solutions were desalted using a disposable PD-10 column (GE Healthcare, USA) with the buffer containing 10 mM Tris (pH 8.0) and 100 mM NaCl as the mobile phase. For membrane proteins (porin and SSS), DDM was included in all buffers during these steps, consistent with their membrane protein nature. The purified proteins were analyzed by SDS-PAGE. The concentrations of the proteins were determined by the bicinchoninic acid (BCA) protein assay kit (Thermo, USA), with bovine serum albumin (BSA) as the standard.

### Enzymatic activity assay

The alginate lyase activity was measured by the ultraviolet absorption spectrometry method (1). Briefly, a 200 μL enzyme reaction system containing enzyme (AlyC2, 14.8 μg/mL; AlyC3, 54 μg/mL; AlyC5, 190 μg/mL; AlyC6, 55 μg/mL; AlyC7, 8.8 μg/mL; AlyC8, 2780 μg/mL; AlyC9, 53 μg/mL; AlyC10, 46 μg/mL; AlyC11, 90 μg/mL) and 2 mg/mL SA in 50 mM Tris-HCl (pH 8.0) and 500 mM NaCl was incubated at 30°C for 10 min. The preheated inactivated enzyme was used as control. The reaction was terminated by boiling the reaction system for 10 min. The increase in the absorbance at 235 nm (*A*_235_), resulting from the release of unsaturated uronic in the mixture, was monitored. One unit (U) of enzyme activity was defined as the amount of enzyme required to produce an increase of 0.1 per minute at 235 nm. The substrate specificity of AlyC2, AlyC3, and AlyC5–C11 was determined at 30°C and pH 8.0 with PM, PG, PMG, and SA as the substrates.

As the substrate (unsaturated monosaccharides) of KdgF spontaneously converts into DEH, the activity of KdgF could not be assayed directly. Instead, we produced unsaturated monosaccharides by exo-alginate lyase AlyC9 and then added KdgF (10.3 mg/mL) to the mixture, which was incubated at 30°C for 2 h. The preheated inactivated enzyme was used as control. The activity of KdgF was tested by monitoring the decrease of *A*_235_ (5).

The DehR activity was assayed as described by Takase and coworkers (51). Briefly, a 200 μL enzyme reaction system containing 20 μL DEH and 20 μL DehR (DehR-1, 19.2 mg/mL; or DehR-2, 3.1 mg/mL) in 50 mM PBS and 200 μM NADPH was incubated at 30°C for 20 min. The preheated inactivated enzyme was used as control. The activity was tested by monitoring the decrease of *A*_340_.

The KdgK activity was tested using a coupled assay, as previously described (52). First, a 200 μL enzyme reaction system containing 100 μg/mL KDG, 20 μL KdgK (16.1 mg/mL), 6 mL ATP 100 mM in 100 mM Tris-HCl (pH 8.0) was incubated at 25°C for 30 min in the dark. Then, 10 μL of NADH (10 mM), 1.5 μL of MgSO_4_ (500 mM), 8 μL of PEP (100 mM), 1.3 μL of lactate dehydrogenase (LDH), and 1 μL of pyruvate kinase (PK) were added to the mixture. The mixture was further incubated at 30°C for 20 min. The preheated inactivated enzyme was used as control. The activity was tested by monitoring the decrease of *A*_340_.

The Eda activity was assayed as previously described (53). Briefly, a 200 μL system containing 420 μM NADPH, 100 μg/mL KDPG, 10 μL of LDH, and 20 mM HEPES was incubated at 40°C for 10 min. Then, 20 μL Eda (6.5 mg/mL) was added to the mixture. The mixture was further incubated at 40°C for 20 min. The preheated inactivated enzyme was used as control. The activity was tested by monitoring the decrease of *A*_340_.

### Analysis of the degradation products of the alginate lyases

The degradation products of the alginate lyases toward SA (AlyC2–AlyC8, AlyC10, and AlyC11) and oligosaccharides (AlyC9) were analyzed by high-performance liquid chromatography (HPLC) with 1 mg/mL saturated mannuronic acid monosaccharide and oligosaccharides at degrees of polymerization (DPs) ranging from 2 to 6 as the standards. The 200 μL reaction mixture containing 1 nmol/mL enzyme, 2 mg/mL substrate, 50 mM Tris-HCl (pH 8.0), and 500 mM NaCl was incubated at 30°C for 24 h. The reactions were terminated by adding 0.4 M trichloroacetic acid (TCA), and the degradation products were analyzed on a Superdex Peptide 10/300 GL column (GE Healthcare, USA) at a flow rate of 0.3 mL/min using 0.2 M ammonium hydrogen carbonate as the running buffer. The eluted glycans were monitored at 210 nm using a UV detector. LabSolutions software was used for online monitoring and data analysis. The degradation products of SA, PM, PG, and PMG by the complex of the secreted alginate lyases, named ∆SA, ∆PM, ∆PG, and ∆PMG, respectively.

### Genetic manipulations

Knockout and complementation of genes *gntr-1*, *gntr-2*, *porin*, *sss*, and *ttt* were performed using vectors pK18mobsacB-Ery (54). Briefly, we amplified the upstream and downstream flanking regions (~0.6 kb) of the target site from the genome of *Vibrio* sp. C42 and then joined them by overlap extension PCR. The 1.2 kb BamHI-HindIII fragments were ligated into the pK18mobsacB-Ery vector. The constructed vectors were then introduced into *Vibrio* sp. C42 by conjugation from an *E. coli* WM3064 strain. The single crossover strains and double-crossover strains were screened by spreading on 2216E containing erythromycin (50 μg/mL) and 2216E containing 12% (wt/vol) L-sucrose, respectively, and the mutant strains *Δgntr-1*, *Δgntr-2*, *Δgntr-1/Δgntr-2*, *Δporin*, *Δsss,* and *Δttt* were constructed. The growth curves of the wild-type strain and its mutant strains in minimal medium containing 0.2% (wt/vol) glucose or 0.2% (wt/vol) alginate at 25°C were compared.

### Isothermal titration calorimetry

Isothermal titration calorimetry (ITC) measurements were performed at 25°C using a MicroCal PEAQ-ITC (Malvern, United Kingdom) in 10 mM Tris–HCl (pH 8.0) containing 100 mM NaCl. The concentration of proteins was 20 μM, and that of the ligands was 5–20 times that of the corresponding protein. The experiment consisted of 13 successive injections of the ligand into the protein solution in the sample cell, with a stirring rate of 750 rpm. Data were analyzed using the Microcal PEAQ-ITC analysis software.

### Crystallization, data collection, and structure determination

TTT-C was concentrated to 12 mg/mL in the buffer containing 10 mM Tris-HCl (pH 8.0) and 100 mM NaCl. Crystallization trials of TTT-C were obtained at 18°C using the sitting-drop vapor diffusion method. TTT-C was mixed with the reservoir solution in a 1:1 (vol/vol) ratio. After 5 days of incubation at 18°C, crystals with diffraction quality were obtained in hanging drops containing 30% (wt/vol) PEG 1500, 0.1 M BICINE (pH 8.5), and 2% (vol/vol) 2-propanol.

X-ray diffraction data were collected on the BL17U1 at the Shanghai Synchrotron Radiation Facility. Initial diffraction data sets were processed using the HKL3000 program (55). Crystal structures of TTT-C were determined by molecular replacement using the CCP4 program Phaser (56). The structure of TTT-C predicted by Alpha-Fold2 (57) was used as the search model. Structure refinement was performed using Coot (58) and Phenix (59).

### Bioinformatics

All available bacterial genomes in the Integrated Microbial Genome database (IMG) were probed for the homologs of the TTT-C protein from *Vibrio* sp. C42 using BLASTP search, with a cut-off value of E value < 1e−50 and identity > 35%. The cut-off values were validated by multiple sequence alignment, structural analysis, and/or biochemical characterization. Subsequently, the NCBI database was used to analyze the genomes of these bacteria to identify AULs containing TTT-C homologs. Genes within the identified AULs were systematically compared, focusing on differences and similarities in alginate lyases, transporters (including SSS and TTT), and regulatory proteins. Gene organization diagrams of the AULs were generated using ChiPlot (https://www.chiplot.online/).

## Data Availability

The genome data of *Vibrio* sp. C42 were deposited in the GenBank database under the accession number JAEKGD000000000.1. The secretome data were deposited in the ProteomeXchange Consortium via the PRIDE under the accession number PXD029037. The RNA-seq data were deposited in NCBI’s Sequence Read Archive (SRA) database with the accession numbers SRR21891194 to SRR21891197. The proteome data were deposited in ProteomeXchange with the identifier PXD037554. TTT-C was deposited in the PDB under the accession code 25HK.

## SUPPLEMENTAL MATERIAL

Supplemental material aem.00368-26-s0001.docx Fig. S1 to S5.

ASM does not own the copyrights to Supplemental Material that may be linked to, or accessed through, an article. The authors have granted ASM a non-exclusive, world-wide license to publish the Supplemental Material files. Please contact the corresponding author directly for reuse.
