# Supplementary material for: A specific tripartite tricarboxylate transporter is involved in alginate utilization by Vibrio sp. C42
Source: Appl Environ Microbiol. 2026 Jun 16;92(7):e00368-26. doi: 10.1128/aem.00368-26 (PMC13390454; doi:10.1128/aem.00368-26)
Supplement: Supplemental material — Fig. S1 to S5. [file aem.00368-26-s0001.docx]

Supplementary Materials

**A specific tripartite tricarboxylate transporter is involved in alginate utilization of *Vibrio* sp. C42**

Xiao-Meng Sun^1,2^, Xiao-Han Wang^1^, Zhao Xue^3^, Peng Wang^2^, Long-Sheng Zhao^1^, Jing-Ping Wang^1^, Ping-Yi Li^1^, Fang Zhao^2^, Shou-Jin Fan^3^, Yu-Zhong Zhang^1,2^, Shu-Yan Wang^1^*, Yu-Qiang Zhang^1^*, Fei Xu^1^*

^1^State Key Laboratory of Microbial Technology, Marine Biotechnology Center, Shandong University, Qingdao, 266237, China

^2^Ministry of Education Key Laboratory of Evolution and Marine Biodiversity, Frontiers Science Center for Deep Ocean Multispheres and Earth System & College of Marine Life Sciences, Ocean University of China, Qingdao 266003, China

^3^Life Science College, Shandong Normal University, Jinan 250014, China

**Running title**: A new TTT-involved alginate utilization pathway

***Address correspondence to**: Shu-Yan Wang, [wangsy0902@126.com](mailto:wangsy0902@126.com); Yu-Qiang Zhang, yqzh2024@sdu.edu.cn; Fei Xu, xufei1028@sdu.edu.cn

**Statements and declarations**

The authors declare no competing interests.


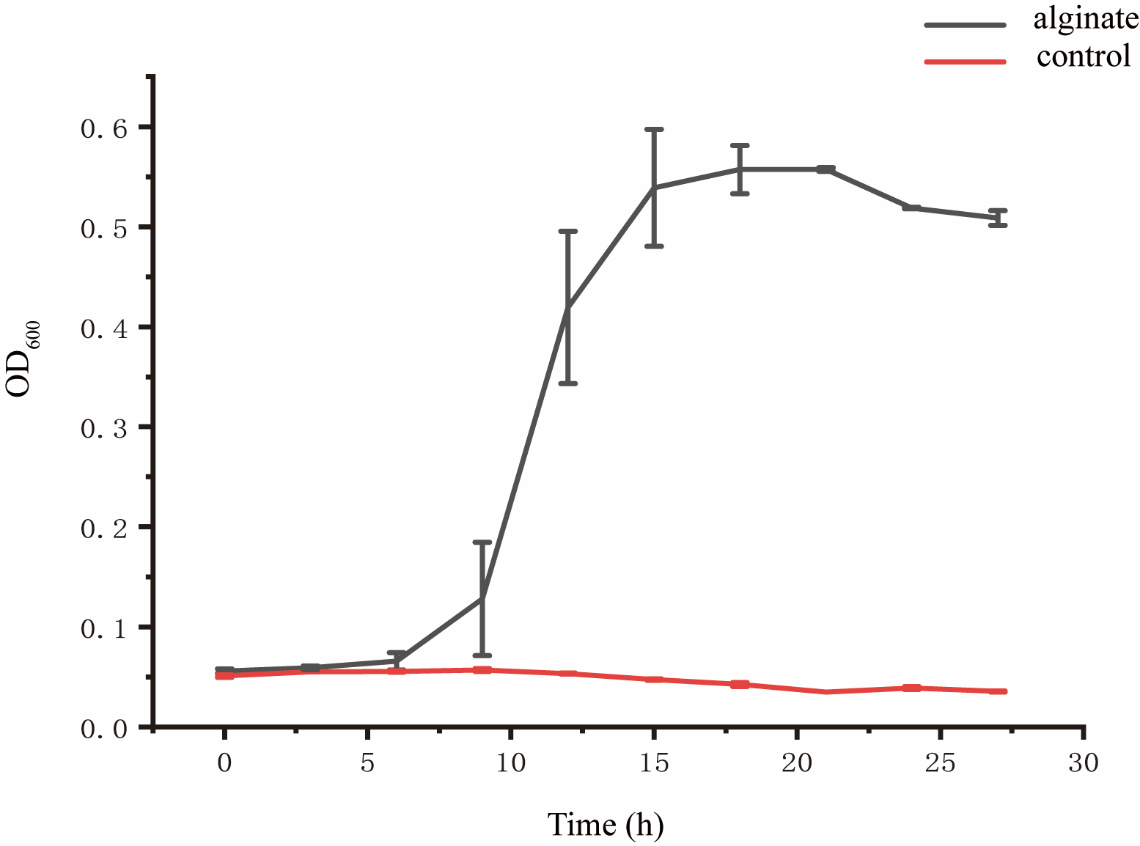


**Figure S1.** **Growth curve of strain *Vibrio* sp. C42 on sodium alginate (SA).** The culture without a carbon source was treated as the control.

**
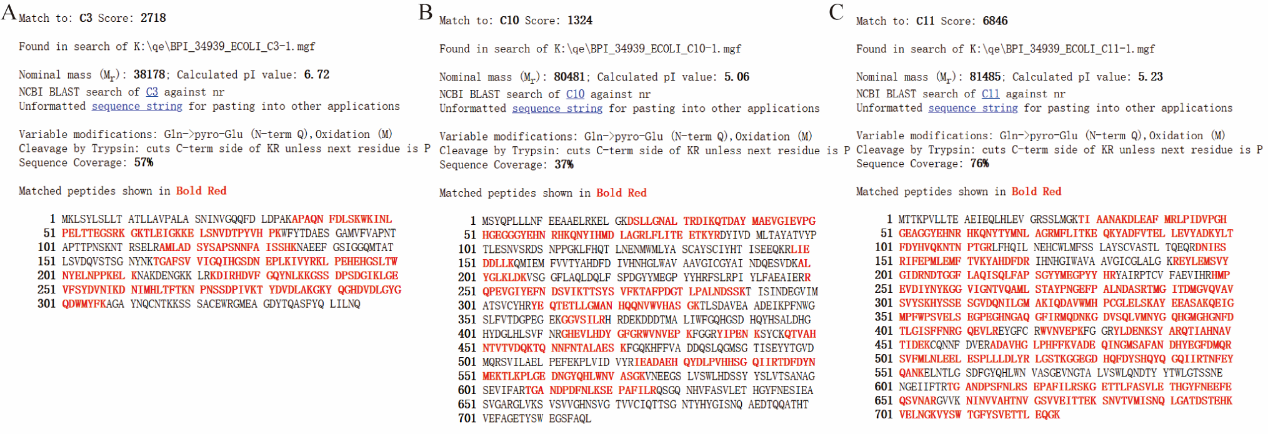
**

**Figure S2. Mass spectrometry identification of AlyC3, AlyC10 and AlyC11.** **Peptide coverage maps of AlyC3 (A), AlyC10 (B) and AlyC11 (C), with matched peptides in bold red.**

**
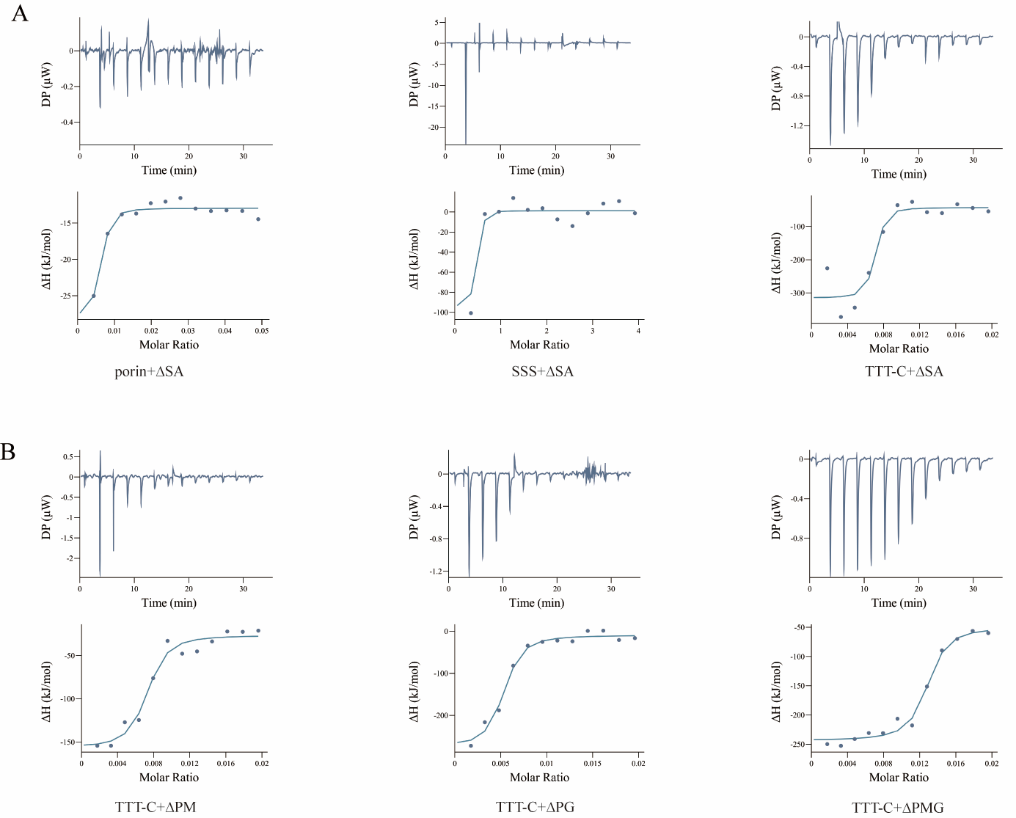
**

**Figure S3. Functional analyses of the transporters.** **(A), Binding capacities of porin, SSS and TTT-C to ∆SA. (B), Binding capacities of TTT-C to ∆PM, ∆PG and ∆PMG.** ITC traces (top) and integrated binding isotherms (bottom) are shown. Experiments were performed in triplicate and representative results are shown.


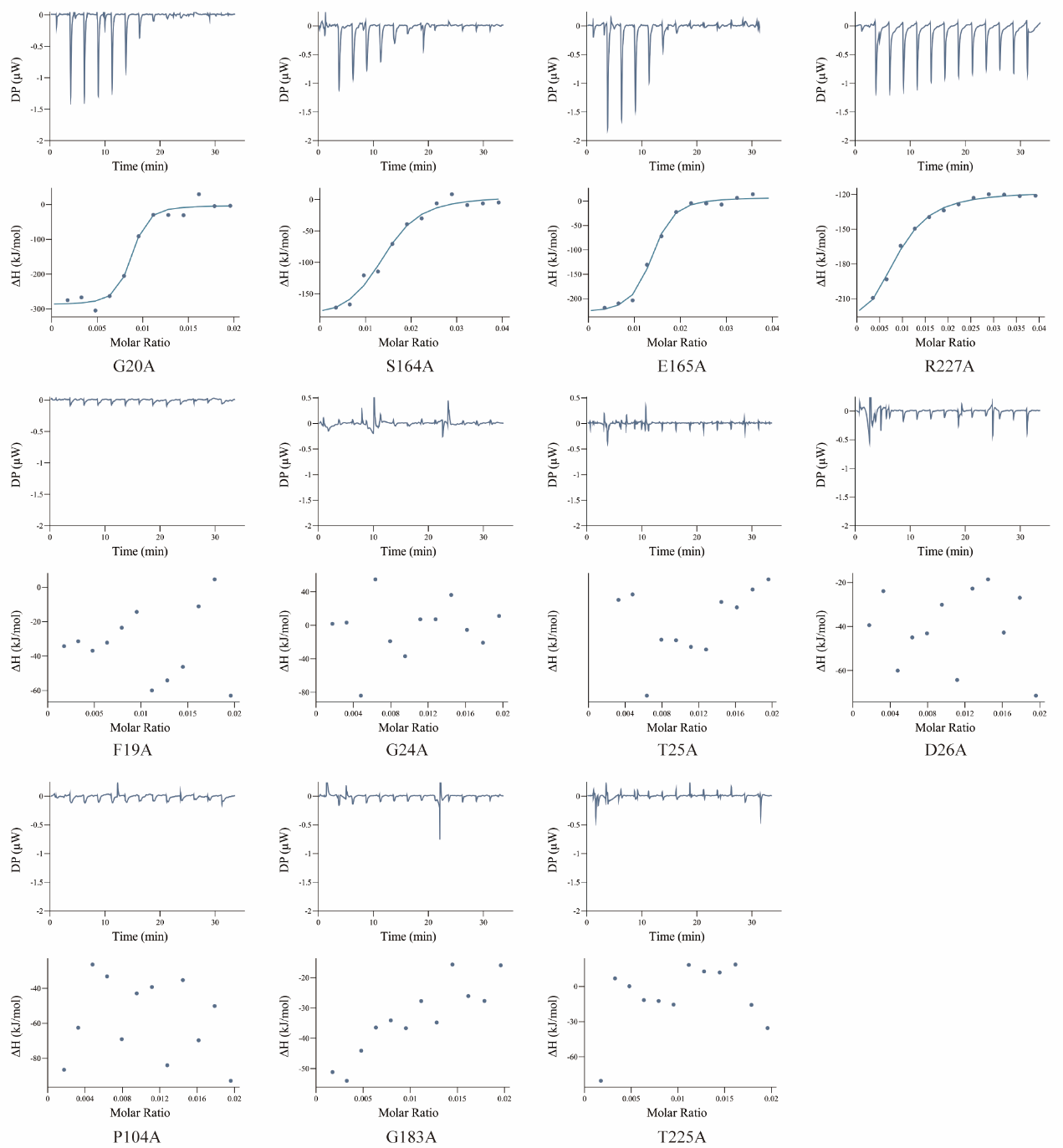


**Figure S4. ITC data for titrations of ∆SA into TTT-C mutants.** ITC traces (top) and integrated binding isotherms (bottom) are shown. Experiments were performed in triplicate and representative results are shown.

**
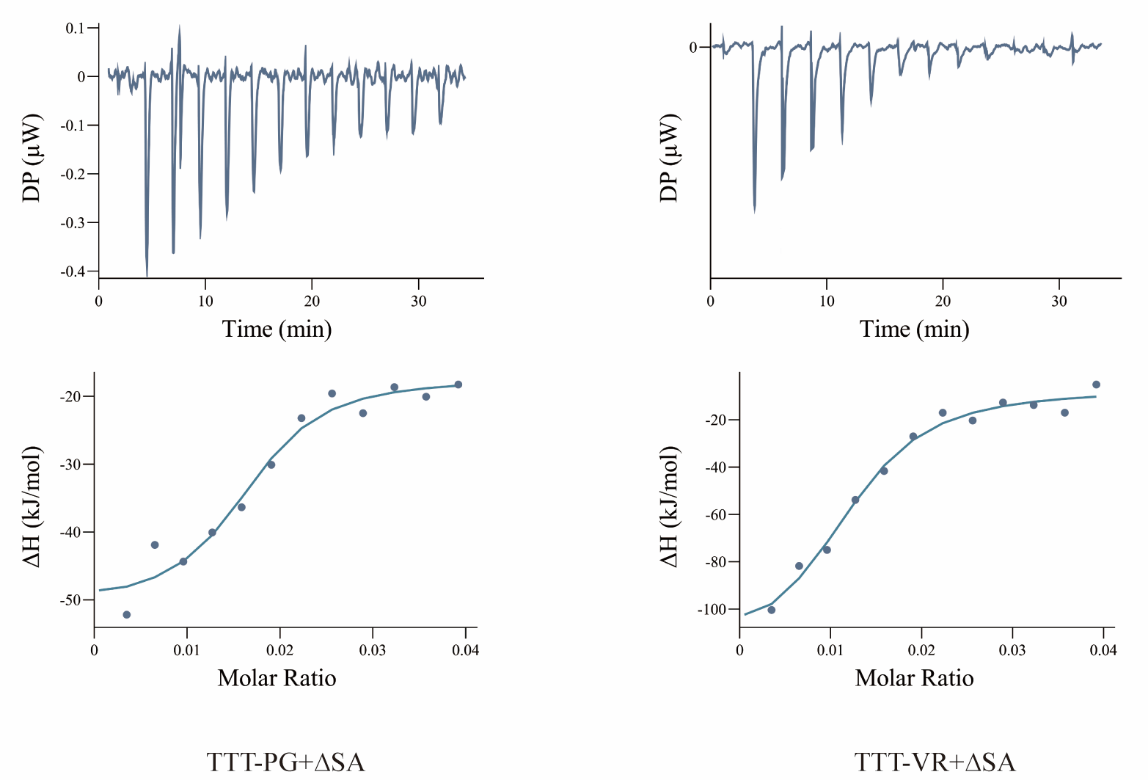
**

**Figure S5.** **ITC data for titrations of ∆SA into TTT-C homologs.** ITC traces (top) and integrated binding isotherms (bottom) are shown. Experiments were performed in triplicate and representative results are shown.
